# Epidemiology of stress fractures among military cadets: a retrospective cohort study at a tertiary military medical center

**DOI:** 10.1097/MS9.0000000000004697

**Published:** 2026-01-22

**Authors:** Abdulaziz Ibrahim Alkhudhayri, Lamia Alkhonain, Abdullah Ahmed Hawsawi, Abdulaziz M. Almuhanna, Maher Alzahrani, Abdullah Mubark Aljeaid, Ahmad Almulla, Hamid Talal Aljohani, Mohammed Saad A. Alhakbani, Abdullah Sami Alharbi, Abdulaziz Saleh Almadi, Abdullah T. Alhaddad

**Affiliations:** a General Practitioner, Buraydah Central Hospital, Qassim, Saudi Arabia; b Orthopedic Department, Prince Sultan Military Medical City (PSMMC), Riyadh, Saudi Arabia; c Orthopedic Clinical Fellow (Trauma & Foot and Ankle Surgery), Division of Orthopedic Surgery, Western University, London, Ontario, Canada; d Orthopedic Department, King Saud Medical City, Riyadh, Saudi Arabia

**Keywords:** cohort study, lower limb injuries, military cadets, orthopedics, Saudi Arabia, stress fracture

## Abstract

**Background:**

Stress fractures are a common overuse injury among physically active populations, yet data on their incidence and characteristics in military trainees remain limited. This study describes epidemiology, anatomical distribution, and management of stress fractures in military students at a tertiary military medical center.

**Methods:**

We performed a retrospective cohort review of 109 male military students diagnosed with stress fractures between January 2020 and August 2025. Demographics, risk factors, fracture sites, and treatment modalities were extracted from electronic medical records and imaging archives; tibial stress fractures were additionally graded using the Fredericson MRI system. Descriptive statistics, including means, standard deviations, frequencies and percentages, and inferential analyses such as chi-square tests, independent-samples t-tests and Mann–Whitney U tests, were conducted using SPSS version 25.0.

**Results:**

The mean age of the cohort was 19.68 ± 1.33 years; 76.1% were under 20 years of age. All fractures involved the lower part of the body, with the tibia most frequently affected (69.7%), followed by the femur (11.0%), foot (8.3%), and hip (8.3%). Anatomically, the right proximal tibia (13.8%) and left proximal tibia (11%) were the most common sites; bilateral injuries accounted for 12.9% of cases. Conservative management was undertaken in 70.6% of cases; 29.4% required surgical intervention, most commonly open reduction and internal fixation. Operative management was significantly associated with fracture location, with hip and femoral fractures more frequently treated surgically than tibial fractures [χ^2^(5, N = 109) = 37.8, *P* < 0.001]. Age and body mass index did not differ significantly between operatively and non-operatively managed patients.

**Conclusion:**

While conservative therapy suffices for most, nearly one-third of fractures required surgical stabilization. These findings support early imaging-based diagnosis, graduated training protocols, and targeted prevention strategies to reduce injury burden and maintain operational readiness.

## Introduction

Stress fractures have been identified as a functional subset of overuse injuries that occur because of repetitive submaximal forces imposed on bones and have been noted in physically active populations such as athletes and military personnel, and account for approximately 10–20% of all injuries seen in sports medicine^[1]^. These injuries are characterized by a distinctive challenge of diagnosis in an early stage, proper management, and long-term prevention^[2]^. While stress fractures have been covered well in general literature, literature describing their epidemiology in military cohorts is somewhat lacking. Quick increases in loads and intensity of physical training due to basic military training are recurrently identified as one of the most significant risk factors for these injuries^[3]^. As defense programs have increased physical demands on military trainees, health care providers have observed a corresponding rise in stress fracture cases^[4]^. However, newer studies indicated that injury surveillance, better physical training strategies, gradual training progression, and focused prevention plans may be slowly reducing the stress fractures^[3,5,6]^. In the past, the management of stress fractures primarily relied on conservative approaches such as rest and activity modification with nutritional optimization^[6]^. In recent years, the management approach has developed to incorporate advanced imaging procedures for early detection or high-risk cases. Surgical intervention is important^[7]^. The main group of patients, consisting of young active-duty male trainees, is trained through strict physical programs exposing them to increased risk of stress fractures.

Most published data on stress fractures in military trainees come from North American, European, and Asian cohorts, and there is a paucity of epidemiological information from Saudi Arabia and the wider Middle Eastern military population^[8,9]^. This retrospective cohort study therefore aims to describe the epidemiological characteristics and anatomical distribution of stress fractures among military students presenting to Prince Sultan Military Medical City (PSMMC).

## Method

Our retrospective cohort study was conducted to examine the epidemiological characteristics and anatomical distribution of stress fractures from January 2020 to August 2025. The institutional review board at PSMMC approved the study. The inclusion criteria were active enrolment as a military student during the study period and an MRI-confirmed diagnosis of stress fracture. The training program for the military students was male-only, so all included cases were male. Exclusion criteria were non-trainee personnel, incomplete clinical or imaging records, and cases in which the clinical and radiological findings did not meet the diagnostic criteria for stress fracture or were judged to be miscoded or misclassified. Each individual was counted once in the main analyses (N = 109); bilateral fractures were recorded as bilateral in the anatomical tables but did not increase the denominator beyond 109. Demographic data including age, gender, smoking status, and relevant medical history were collected, along with injury-specific data such as anatomical site, side, and extent of fracture involvement. All patients first underwent clinical assessment and plain radiographs of the symptomatic region. MRI was not performed routinely but was requested when plain radiographs were normal or equivocal in the setting of high clinical suspicion, when a high-risk site such as the femoral neck, hip, or proximal tibia was involved, or when symptoms persisted despite an initial period of rest. According to institutional protocol, all clinically suspected stress fractures meeting these criteria underwent MRI. Radiological reports and MRI findings were reviewed by experienced orthopedic consultants. Tibial stress fractures were graded using the Fredericson MRI grading system, whereas stress fractures at other anatomical sites were confirmed on MRI but not formally graded. Lesions showing only diffuse bone marrow edema without discrete cortical or periosteal change were considered bone stress reactions rather than stress fractures and were not analyzed as fractures in this cohort. Details regarding the type of treatment, whether conservative or surgical, and the specific operative interventions performed were extracted and recorded.

### Statistical analysis

All analyses were performed using IBM SPSS Statistics, version 25. Categorical variables, such as affected bone, anatomical site, and treatment modality, were summarized as frequencies and percentages. Continuous variables, including age and body mass index (BMI), were summarized as means and standard deviations. The various management categories were regrouped into a binary treatment variable with two levels: non-operative (conservative management) and operative (any surgical procedure). The association between affected bone and treatment strategy was examined using a chi-square test of independence, and effect size was quantified with Cramér’s V. The distribution of age and BMI was assessed using the Shapiro–Wilk test. When the assumption of normality was considered acceptable, comparisons between treatment groups were made using independent-samples t-tests. When normality was not met, comparisons were made using the Mann–Whitney U test. A two-sided *P*-value of less than 0.05 was considered statistically significant.

**HIGHLIGHTS**

- Design & cohort: Retrospective cohort at Prince Sultan Military Medical City, Riyadh; n = 109 male cadets; mean age 19.68 ± 1.33 years.
- Anatomical distribution: Tibia 69.7% (76/109); femur 11.0% (12/109); foot 8.3% (9/109); hip 8.3% (9/109); pelvis 1.8% (2/109); fibula 0.9% (1/109).
- Management pattern: Conservative 70.6%; surgical 29.3% overall. Principal procedures included open reduction and internal fixation (ORIF) 11.0% and closed reduction with intramedullary nailing 10.1%, with additional isolated procedures reported (e.g., femoral neck system 0.9%, one bilateral ORIF 0.9%).
- Key findings: Lower-limb stress fractures predominantly involve the tibia; clear concentration in load-bearing bones.
- Clinical relevance: Data support targeted load-management and conditioning, early imaging-guided diagnosis, and structured rehabilitation pathways to minimize training disruption.

### Guidelines adherence

This manuscript aligns with the TITAN checklist for transparency in the reporting of Artificial intelligence and the work has been reported in line with the STROCSS criteria^[10,11]^.

## Result

### Study population and demographics

A total of 109 military students were included in this retrospective cohort study conducted at our hospital. All participants were male (100%). The mean age of the cohort was 19.68 years [standard deviation (SD) = 1.326], primarily including young adulthood. The majority of participants (76.1%) were under 20 years of age, while the remaining 23.9% were aged 20–24 years. For BMI, the mean (SD) was 22.5 (3.1). For risk factors, only three participants (2.8%) were smokers; the remaining 106 participants (97.2%) were non-smokers. Past medical history showed that two participants (1.8%) had a diagnosis of bronchial asthma and the remaining 107 participants (98.2%) had no notable past medical conditions. All 109 participants (100%) were diagnosed with military-related stress fractures. When patients were grouped by treatment strategy, age and BMI did not differ significantly between those managed non-operatively and those treated operatively. The mean (SD) of age was 19.6 (1.1) years in the non-operative group and 20.0 (1.7) years in the operative group (Mann–Whitney U = 1063.0, *P* = 0.25). The mean (SD) of BMI was 22.6 (3.4) kg/m^2^ in the non-operative group and 22.3 ± 2.5 kg/m^2^ in the operative group (t (107) = 0.40, *P* = 0.69). These comparisons are summarized in Tables 1 and 2.

**Table 1:** Demographic data

Total number	109
Gender	
Male	100 (100%)
Female	0 (0%)
Average age [mean (SD)]	19.68 (1.326)
BMI [mean (SD)]	22.5 (3.1)
Age groups (years)	
< 20	83 (76.1%)
20–24	26 (23.9%)
Risk factor	
Smoker	3 (2.8%)
None	106 (97.2%)
Past medical history	
Bronchial asthma	2 (1.8%)
None	107 (98.2%)
Military stress fracture	109 (100%)

**Table 2:** Site of the injury

Site of the injury	Number	%
Lower limb affected	109	100
Affected bone	Number	%
Femur	12	11
Fibula	1	0.9
Foot	9	8.3
Hip	9	8.3
Pelvis	2	1.8
Tibia	76	69.7

### Site of injury and anatomical distribution

All 109 stress fractures involved the lower limb or pelvis. The tibia was the most commonly affected bone (69.7%, n = 76), followed by the femur (11.0%, n = 12), foot bones (8.3%, n = 9), and hip (8.3%, n = 9), with fewer fractures in the pelvis (1.8%, n = 2) and fibula (0.9%, n = 1), as shown in Table 3. At anatomical-site level, stress fractures clustered predominantly in the proximal and shaft segments of the tibia. The right proximal tibia (13.8%, n = 15), left proximal tibia (11.0%, n = 12), and left tibial shaft (10.1%, n = 11) were the most frequent locations, with additional cases in the tibial plateau and midshaft. Bilateral involvement accounted for 12.9% of cases, most commonly affecting proximal or midshaft tibia. Less common sites such as the sacrum, navicular, cuneiform, and talus each contributed small proportions to the overall distribution (Table 4).

**Table 3:** Anatomical site of injury

Anatomical site of injury	Number	%
Bilateral distal femur	1	0.9
Bilateral femoral neck	1	0.9
Bilateral midshaft tibia	3	2.8
Bilateral proximal tibia	6	5.5
Bilateral tibial plateau	3	2.8
Left calcaneus	1	0.9
Left distal femur	3	2.8
Left distal tibia	3	2.8
Left femoral neck	2	1.8
Left femoral shaft	1	0.9
Left Lisfranc	3	2.8
Left navicular	2	1.8
Left proximal femur	1	0.9
Left proximal tibia	12	11.0
Left tibial plateau	5	4.6
Left tibial shaft	11	10.1
Right cuneiform	1	0.9
Right distal femur	4	3.7
Right distal tibia	2	1.8
Right femoral head	1	0.9
Right femoral neck	5	4.6
Right femoral shaft	2	1.8
Right mid-shaft fibula	1	0.9
Right midshaft tibia	8	7.3
Right proximal tibia	15	13.8
Right talus	2	1.8
Right tibial plateau	8	7.3
Sacrum	2	1.8
Total	109	100.0

**Table 4:** Treatment modalities for fracture management

Treatment category	n	%
Conservative management (total)	77	70.6
Restriction of heavy activity	39	35.8
WBAT and avoiding exercise	14	12.8
NWB backslab, crutches	13	11.9
NWB with crutches	10	9.2
PWB and avoiding heavy exercise	1	0.9
Operative management (total)	32	29.4
CRIF with intramedullary nailing (IMN)	11	10.1
ORIF	12	11.0
ORIF by plate and screws	3	2.8
CRIF with cannulated screws	3	2.8
Dynamic hip screw (DHS)	1	0.9
Femoral neck system (FNS)	1	0.9
ORIF bilaterally	1	0.9
Total	109	100.0

### Treatment modalities for fracture management

Conservative management was undertaken for most military students with stress fractures (n = 77, 70.6%), typically through restriction of heavy activity, weight-bearing as tolerated with activity modification, or non-weight-bearing with backslab and crutches, as detailed in Table 5. The remaining 32 patients (29.4%) underwent surgery. Most operative procedures involved open reduction and internal fixation or intramedullary nailing, with smaller numbers treated using plate-and-screw constructs, dynamic hip screw or femoral neck system (Table 5). The proportion of operative management varied substantially by affected bone: femoral fractures were treated operatively in 8 of 12 cases (66.7%), hip fractures in 9 of 9 cases (100%), whereas only 12 of 76 tibial fractures (15.8%) underwent surgery; pelvic and fibular fractures were managed non-operatively in all cases, and 3 of 9 foot fractures (33.3%) were treated surgically. The association between affected bone and treatment strategy was statistically significant [χ^2^(5, N = 109) = 37.8, *P* < 0.001; Cramér’s V = 0.59], indicating a large effect size (Table 6).

**Table 5:** Age and BMI according to treatment strategy

Variable	Non-operative (n = 77)	Operative (n = 32)	*P* value
Age, years, mean (SD)	19.6 ± 1.1	20.0 ± 1.7	0.25^a^
BMI, kg/m^2^, mean (SD)	22.6 ± 3.4	22.3 ± 2.5	0.69^b^

^a^ Mann–Whitney U test.

^b^ Independent-samples t test.

**Table 6:** Treatment strategy according to affected bone

Affected bone	Non-operative n (%)	Operative n (%)	Total n (%)
Tibia	64 (84.2)	12 (15.8)	76 (69.7)
Femur	4 (33.3)	8 (66.7)	12 (11.0)
Hip	0 (0.0)	9 (100.0)	9 (8.3)
Foot	6 (66.7)	3 (33.3)	9 (8.3)
Pelvis	2 (100.0)	0 (0.0)	2 (1.8)
Fibula	1 (100.0)	0 (0.0)	1 (0.9)
Total	77 (70.6)	32 (29.4)	109 (100.0)

## Discussion

This retrospective study characterized stress fractures in 109 young male military students, revealing a predominance of tibial involvement (69.7%), a conservative first treatment approach (70.6%), and a mean age of 19.7 years. These findings align closely with previous military cohorts and emphasize the need for targeted prevention and management strategies in high intensity training environments.

### Epidemiology and demographics

The mean age of 19.7 years in our cohort mirrors the young-adult demographic commonly reported in military stress fracture studies. A prospective study found a 31% incidence of stress fractures among new recruits, predominantly affecting tibial or femoral shafts in an 18–20-year-old male population^[12]^. In Indian Border Security Force cadets, the incidence reached 15% during basic training, again with young males bearing the highest burden^[13]^. This concentration of stress fractures in late-adolescent male trainees suggests that peak training load during initial military training coincides with a vulnerable period for bone adaptation, highlighting the need for close monitoring in this age group^[15]^. Our cohort therefore reflects a homogeneous group of full-time male cadets training in a standardized residential program at a single national military center, which provides a relatively uniform exposure profile compared with more heterogeneous military populations.

### Anatomical distribution

Our finding that 69.7% of stress fractures occurred in the tibia is consistent with multiple cohorts reporting tibial predominance: 86.5% in Indian recruits with the proximal third most affected^[13,16]^, and more than 80% tibia/femur involvement in Israeli recruits^[12]^. In our cohort, the right proximal tibia (13.8%) and left proximal tibia (11%) were the most frequent anatomical sites, consistent with other reports in which the tibia were identified as hotspot, accounting for 52.11% of stress fractures, respectively^[14]^.) Less common sites such as the sacrum, navicular, and cuneiform bones (≤1.8%) were also reported in a U.S. military study, reinforcing the spectrum of lower limb involvement^[8]^. The predominance of proximal and shaft tibial lesions is consistent with high cyclic loading during marching and running and supports focusing screening and preventive measures on the tibia in young military trainees.

### Potential risk factors and training context

BMI was within the normal range (mean 22.5 kg/m^2^) and did not differ significantly between operatively and non-operatively managed patients, so within this narrow range we did not observe a clear pattern linking BMI to management pathway. The very low prevalence of smoking 2.8 % and asthma 1.8 %, also means that these variables cannot be interpreted as established risk factors in our data. Because of the retrospective design, detailed information on training intensity, footwear and nutritional status was not available; however, the combination of high-volume load-bearing exercise, rapid escalation of training load and possible variation in conditioning practices is likely to influence stress fracture risk in this setting and should be examined prospectively in controlled studies^[18]^.

### Treatment modalities

Conservative management remains the mainstay for low risk or non-displaced stress fractures. In our study, 70.6% of patients underwent non-operative care, paralleling BSF cadet data where the majority healed without surgery^[16]^. However, 29.4% required surgical intervention, predominantly open reduction and internal fixation (ORIF). The selective use of intramedullary nailing and cannulated screws in our cohort reflects an emergent trend favoring early stabilization in cases prone to delayed union or complete fracture^[7,17]^. Our epidemiological and anatomical distributions largely corroborate global data on military stress fractures. The high incidence in the proximal tibia and the predominance of young male demographics have been consistent themes across studies in Israel and India^[12,16]^. The conservative first paradigm also parallels established guidelines, with surgical intervention reserved for displaced or recalcitrant cases^[2,7,17]^. Notably, our surgical rate (29.4%) appears higher than in many published cohorts, which may reflect differences in training demands or early MRI-based detection prompting earlier operative management^[1,2,7,17]^. In our cohort, the choice of operative versus conservative management was strongly influenced by fracture site, with hip and femoral fractures much more likely to undergo surgery than tibial fractures, consistent with their higher risk profile. Our higher surgical rate therefore appears to reflect both the case mix and institutional practice: a relatively large proportion of high-risk hip and femoral fractures, together with early MRI-based identification of unstable lesions, may have lowered the threshold for operative stabilization compared with cohorts dominated by low-risk tibial stress fractures. In addition, the military training context, in which cadets are expected to return to full duty promptly, may have further favored operative management. The combination of routine MRI confirmation of suspected stress injuries and ready access to subspecialist orthopedic care at PSMMC is also likely to influence decision-making thresholds and may help explain the relatively high operative rate observed in this cohort. Age and BMI were similar between patients managed operatively and non-operatively, suggesting that in this cohort treatment decisions were driven more by fracture characteristics and institutional practice than by baseline anthropometric factors.

### Implications for military training

These findings underscore the importance of structured prevention strategies, including graduated training loads, footwear optimization, and routine bone stress screening – especially MRI for high-risk anatomical sites. Early identification and risk stratification may reduce progression to complete fractures and the need for surgical intervention.

## Limitations

Limitations include the retrospective design, the inclusion of only symptomatic cadets who sought medical attention and were recorded in hospital systems, and the exclusive focus on male trainees, so asymptomatic or self-managed stress reactions may have been missed. Inter-observer variability in the Fredericson MRI grading system, which we used to grade tibial stress fractures according to the extent of periosteal and marrow edema and the presence of a fracture line, was not assessed, and long-term functional outcomes remain to be evaluated. Follow-up and outcome measures were not standardized, so data on time to healing, complications, return to training, and use of orthoses were incompletely documented and could not be analyzed. Vitamin D status, diet, and bone mineral density were not systematically recorded, so underlying bone health could not be assessed, and as a single-center study in a relatively small, homogeneous male cohort, the generalizability of our findings is limited. In addition, we did not include a comparison group of trainees without stress fractures as it is cohort study, so we were unable to determine whether clinical characteristics such as smoking status or asthma differ from the underlying cadet population; as such, these variables should not be interpreted as established risk factors in this study.

## Conclusion

Stress fractures in military students predominantly affect young male trainees and most commonly involve the tibia, with most cases healing under conservative management and a minority requiring surgery. These findings support early detection pathways and tailored treatment to minimize time away from training. Military institutions should integrate ongoing surveillance for bone stress injuries with graduated training programs and structured recovery protocols to reduce injury burden and maintain operational readiness.

## Data Availability

Available upon reasonable request.

## Research registration unique identifying number (UIN)

Prince Sultan Military Medical City Scientific Research Center Institutional Review Board with registration number: HP-01-R079.
